# Influence of the Traverse Speed of the Stylus Tip on Changes in the Areal Texture Parameters of Machined Surfaces

**DOI:** 10.3390/ma17205052

**Published:** 2024-10-16

**Authors:** Pawel Pawlus, Rafal Reizer, Wiesław Żelasko

**Affiliations:** 1 Faculty of Mechanical Engineering and Aeronautics, Rzeszow University of Technology, Powstancow Warszawy 8 Street, 35-959 Rzeszow, Poland; 2 Institute of Materials Engineering, College of Natural Sciences, University of Rzeszow, Pigonia Street 1, 35-310 Rzeszow, Poland; rreizer@ur.edu.pl; 3 Faculty of Mechanics and Technology, Rzeszow University of Technology, Kwiatkowskiego Street 4, 37-450 Stalowa Wola, Poland; w.zelasko@prz.edu.pl

**Keywords:** traverse speed, stylus flight, surface texture parameters

## Abstract

Measurements of areal (3D) surface texture using optical methods are very popular because of the short measurement time compared to the stylus tip technique. However, they are very sensitive to measurement errors. In some cases, optical measurements are not recommended. The stylus measurement method is well known and can be the reference technique for surface texture measurement. The main disadvantage is the long measuring time. This time can be shortened using higher speeds of measurement. The effect of the speed of the measurement of stylus profilometer on changes in surface texture parameters was studied. Fifty surface topographies were measured using the stylus profilometer at speeds 0.5, 1, 2, 3, 4, and 5 mm/s in the same places. Surfaces after lapping, polishing, grinding, milling, laser texturing, and two-process random surfaces were measured and analyzed. Changes in parameters caused by the increase in the traverse speed depend on the characteristics and parameters of the surfaces. The random surfaces changed more than the deterministic ones. The increase in the traverse speed from 0.5 to 1 mm/s caused small changes in the parameters.

## 1. Introduction

The stylus technique is commonly used to characterize roughness profiles. Stylus profilometers were used to measure areal surface topography. This technique is known [1,2]. The typical measurement errors produced by this method are related to the interaction between the tip of the stylus and the measured surface, known as mechanical filtration [3,4,5,6]. These errors should be minimized by decreasing the radius of the tip of the stylus. The main problem in measuring the topography of the areal surface topography with the stylus technique is the long measurement time. This is related to the restricted measurement speed. Therefore, stylus profilometers were recently replaced by optical methods in the measurement of areal surface texture [7]. However, optical methods are very sensitive to measurement errors. These errors can be caused by many factors such as presences of spikes, non-measuring points, and high-frequency noise.

In some cases, the stylus technique must be used for the measurement of the topography of the areal surface, especially for surfaces that reflect light poorly. Moreover, the stylus tip technique is well known and may represent a reference technique for surface texture measurement.

Topography distortion can occur when the tip of the stylus loses contact with the measured surface. This behavior can take place because of a rapid impulse, for example when the tip encounters a sharp peak. This is called a stylus flight. The high peaks in the measured surface prove the presence of stylus flight [8]. The negative slope became more gentle due to the flight of the stylus [2]. Pawlus and Smieszek [9] and Gorka [10] found that the flight of the stylus caused significant changes in parameters related to the peaks and slope. They analyzed the results of surface topography parameters using the Talyscan 150 stylus profilometer. Gorka [10], in addition to the one-process textures, studied changes in parameters of two-process surfaces. Davinci et al. [11] obtained an increase in the amplitude parameters Sa, Sq and Sz with scanning speeds of 2 and 3 mm/s, compared to a speed of 0.5 mm/s. The maximum changes were less than 2%. They measured periodic surfaces characterized by the Sq parameter of 7 µm. Swiderski [12] analyzed the effects of traverse speed on changes in profile parameters of roughness patterns after lapping, milling, grinding, and turning. The sampling interval was 0.125 µm. He obtained the highest changes in rms. slope among profile parameters caused by increasing stylus speed compared to the reference speed of 0.1 mm/s for a traverse speed of 2 mm/s. The highest increases depended on the measured sample, and in some cases were higher than 100%.

Surface topography measurements are typically performed with a traverse speed of 0.5 mm/s, while the fastest speed generally used is 1 mm/s [2]. However, Morrison developed a special device for measuring small surface areas with a scanning speed of 5 mm/s [13].

The contact of the stylus with the measured surface was modeled and analyzed by many researchers. Damir [14] determined the appropriate stiffness of the spring and the initial deflection of the spring to prevent separation between the stylus and the surface. McCool [15] evaluated the combined sources of errors due to the finite size of the stylus tip and stylus kinematics. However, the results of the modeling presented in [14,15] were not experimentally verified, contrary to the models developed by Song and Vorburger [8] and Pawlus and Smieszek [9]. Tian et al. [16] developed a model to study the characteristics of the stylus instruments. This model incorporated both tip size and tip dynamics, considering stylus flight. The novel dynamic model of stylus contact developed by Tian et al. [17] considered Hertzian contact stiffness and damping forces. They suggested applying damping ratios much higher than conventionally used, since the damping caused a delay or suppressed stylus flight. An increase in the damping ratio was previously successfully applied in [18]. Fang et al. [19] modeled the dynamic behavior of the stylus probing system. This method did not consider adhesion, friction, or elastic deformation.

The stylus method should be used in some cases when it is difficult to measure using optical methods. Because of the known characteristics, it can be treated as a reference method. Stylus reference measurements of machined surfaces are important in validating optical measurements. However, the measurement time of the topography of the areal surface is long. It can be shortened using a higher traverse speed. Reduced time due to increased transverse speed can lead to a decrease in the cost of measurement. But high speed corresponds to stylus flight. The force, damping constant of the stylus, and character of the measured surface affect this phenomenon.

The authors of only a few articles [9,10,11] studied the effects of the speed of the stylus profilometer on changes in the parameters of the areal surface texture. In these works, only changes of selected parameters were considered, a small number of surfaces of selected types were analyzed, and measurements were made using a small number of scanning speeds. This work tries to fill these gaps. The novelty of this work is to find a relation between the parameters characterizing the surface profile along the measurement direction and changes in surface texture rms. slope Sdq due to an increase in the traversing speed. This relation should be valid for surfaces after various machining processes.

## 2. Materials and Methods

Fifty surfaces of 43CrMo4 steel, of 40 HRC hardness, were measured using a Talysurf i series stylus profilometer (Taylor Hobson Ltd., Leicester, UK) equipped with a tip radius of 2 µm; the stylus force was 1 mN. Surface topographies after lapping/polishing (12 surfaces), grinding (12 surfaces), milling (10 surfaces), laser texturing (9 surfaces) and two-process random surfaces [20] after abrasive blasting and lapping (7 surfaces) were measured and analyzed. Milling, grinding, polishing, lapping, and abrasive blasting are typical machining processes. Laser texturing leads to improvements in the tribological properties of machine elements [21,22]. Two-process random textures had traces of two random processes. They are characterized by better functional performance than one-process textures [20,23,24].

The measured surface textures had a varied character. Therefore, the results obtained should be representative and generalizable. Each surface was measured with speeds of 0.5, 1, 2, 3, 4 and 5 mm/s. The back movement was 1 mm/s. The sampling intervals were 2 µm in the measurement direction (the sampling interval should not be less than the tip radius [1]) and 5 µm in the perpendicular direction. The assessment length in the measurement direction was 2 mm and in the perpendicular direction it was 1 mm. Prior to the calculation of parameters, the surfaces were only leveled (for a polished surface, form was eliminated using a polynomial of the second degree). Digital filtration was not used. The following parameters contained in the ISO 25178 standard [25,26,27] were analyzed: rms. height Sq, skewness Ssk, kurtosis Sku, maximum peak height Sp, maximum pit height Sv, maximum surface height Sz, arithmetic mean height Sa, autocorrelation length Sal, texture–aspect ratio Str, rms. slope Sdq, developed interfacial area ratio Sdr, density of peaks Spd, and arithmetic mean peak curvature Spc. Sq, Sa, Sp, Sv and Sz characterize the amplitude, Ssk and Sku reflect the shape of the ordinate distribution [28], Sal and Str are spatial surface properties, Sdq and Sdr are hybrid parameters, and Spd and Spc are related to peaks [25].

The results obtained using the smallest speed of 0.5 mm/s were the reference results. This speed is commonly used in the measurement of surface roughness [1].

Surface topographies were measured from the left to right sides of the presented figures.

## 3. Results and Discussion

In the tables and figures, the results of the measurement of representative surfaces obtained after the same treatment are presented. Similar tendencies of parameter changes due to increasing traverse speed were obtained for other surfaces.

Table 1 presents selected texture parameters of surface A after abrasive blasting. Standard deviations of repetitive measurements at the same place of the selected parameters are given.

Changes in most of the parameters due to an increase in measurement speed were greater than scatters of the parameters due to repetitions of measurement. The highest relative scattering of roughness values occurred for Sdc and Sku parameters. The spatial parameters Sal and Str were stable. A tendency was found whereby the scattering of parameters was higher when changes of parameters due to the increase in the measurement speed were greater. A similar trend occurred for other measured surfaces.

**Figure 1:**
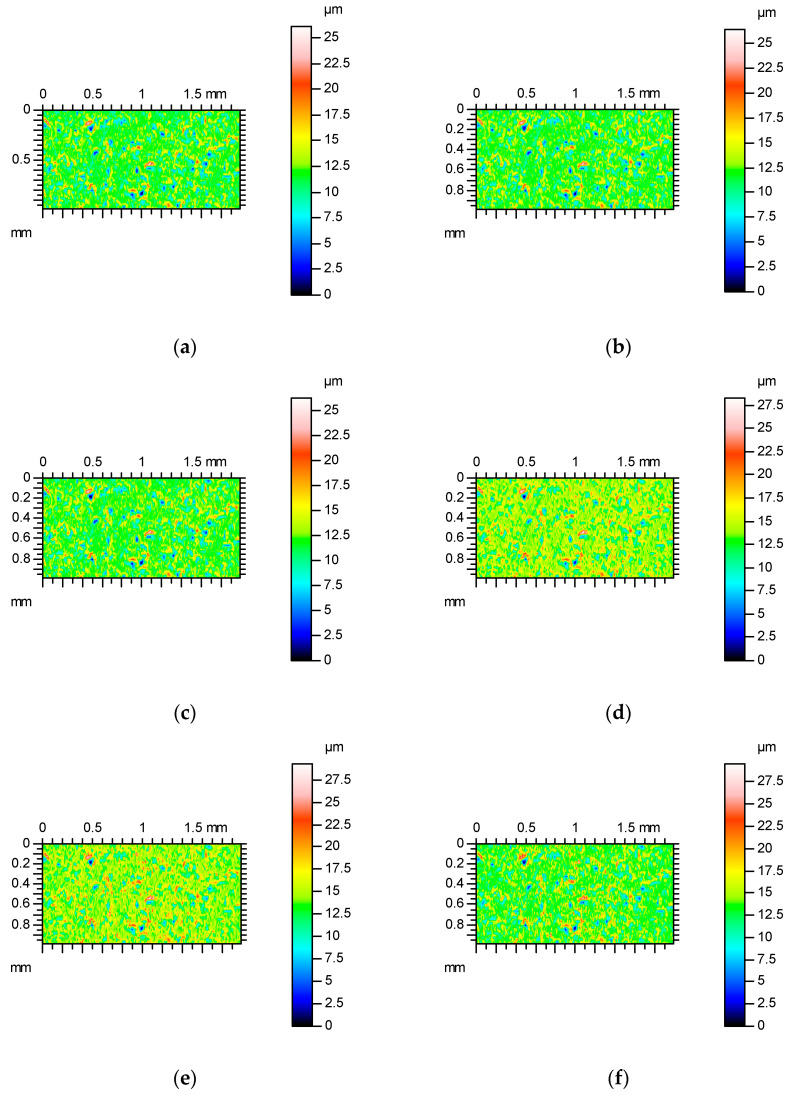
Contour plots of surface A after abrasive blasting after measurements with various speeds: (**a**) 0.5 mm/s, (**b**) 1 mm/s, (**c**) 2 mm / s, (**d**) 3 mm/s, (**e**) 4 mm/s and (**f**) 5 mm/s.

**Figure 2:**
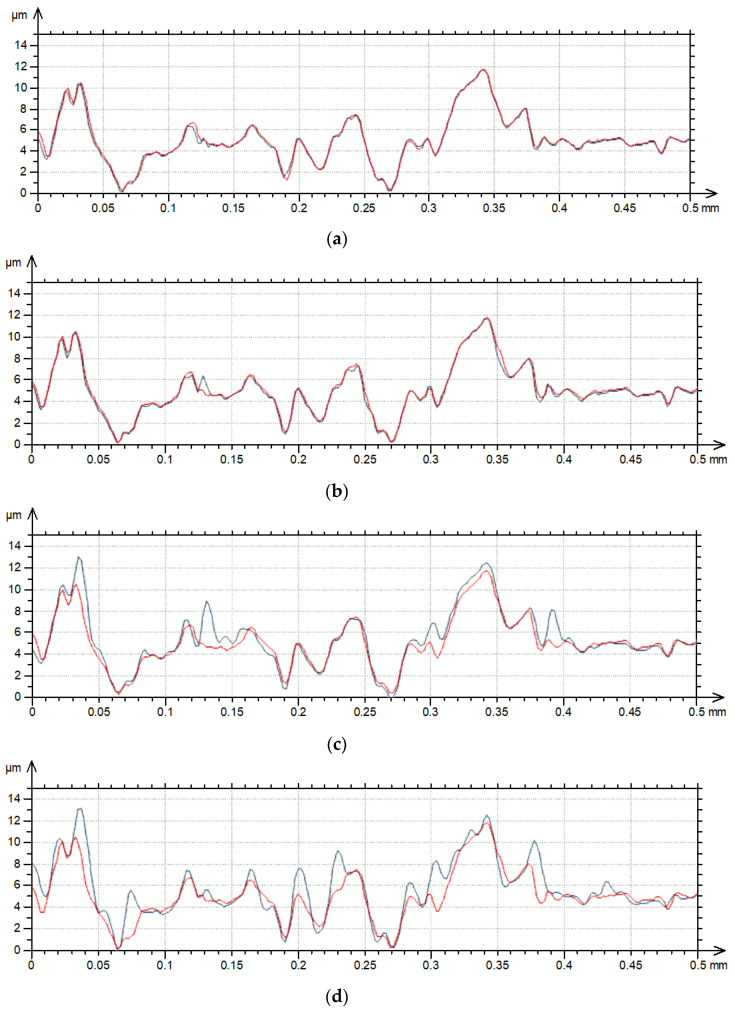

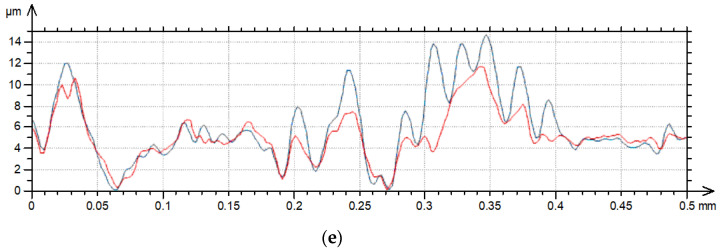
Profile details of surface A after measurements with various speeds: (**a**) 0.5 and 1 mm/s, (**b**) 0.5 and 2 mm/s, (**c**) 0.5 and 3 mm/s, (**d**) 0.5 and 4 mm/s, (**e**) 0.5 and 5 mm/s; red color—0.5 mm/s speed, blue color—higher speeds.

Figure 1 shows contour plots, and Figure 2 shows profile details of this texture.

The analysis in Table 1 shows that the speed of the increase in the traverse speed led to an increase in the amplitude parameters that characterize the mean height Sa and Sq, as well as the Sp of the surface A after abrasive blasting. The greatest increase occurred for the Sa parameter (about 12%). These parameters increased as the measurement speed increased. The other amplitude parameters Sz and Sv also increased with the measurement speed to a speed of 4 mm/s, and then decreased. The greatest increase occurred for the Sv parameter and was 25%. No clear trend of Ssk skewness and Sku kurtosis changes occurred. The spatial parameters Sal and Str were constant. The increase in measurement speed caused an increase in the peak density; the greatest increase of 43% occurred with a measuring speed of 3 mm/s. Changes in hybrid parameters are the consequence of changes in amplitude and in peak density. The maximum increase in Sdq and Sdr occurred with a measurement speed of 4 mm/s and amounted to 20 and 40%, respectively. An increase in measurement speed led to an increase in the mean peak curvature Spc; the greatest increase of 34% occurred for a speed of 4 mm. However, a further increase in the measuring speed to 5 mm/s caused a decrease in the Spc parameter.

Figure 1 proves that the surface amplitude increased with measuring speeds of 3, 4 and 5 mm/s. The increase in measuring speed led to an increase in height and the presence of additional peaks (not really present on the surface)—Figure 1. These peaks are also visible on the contour plots presented in Figure 2 for measuring speeds of 3, 4 and 5 mm/s.

**Figure 3:**
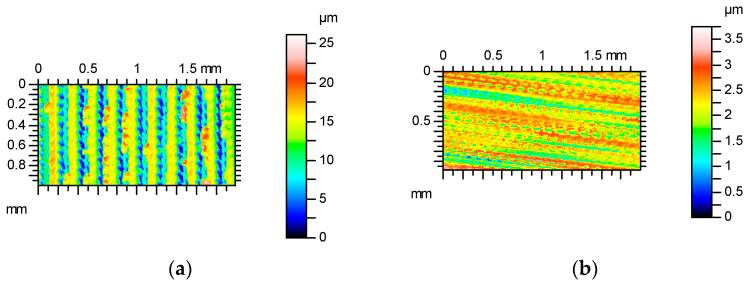

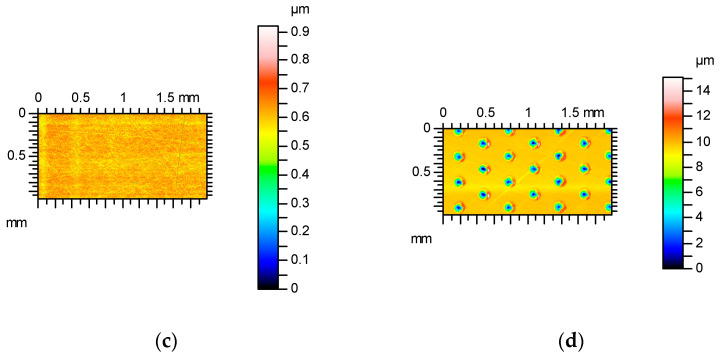
Contour plots of surfaces B (**a**), D (**b**), E (**c**), and F (**d**) after measurements with speeds of 0.5 mm/s.

Figure 3 presents contour plots of surfaces B, D, F, and E, measured with the smallest speed of 0.5 mm/s.

**Figure 4:**
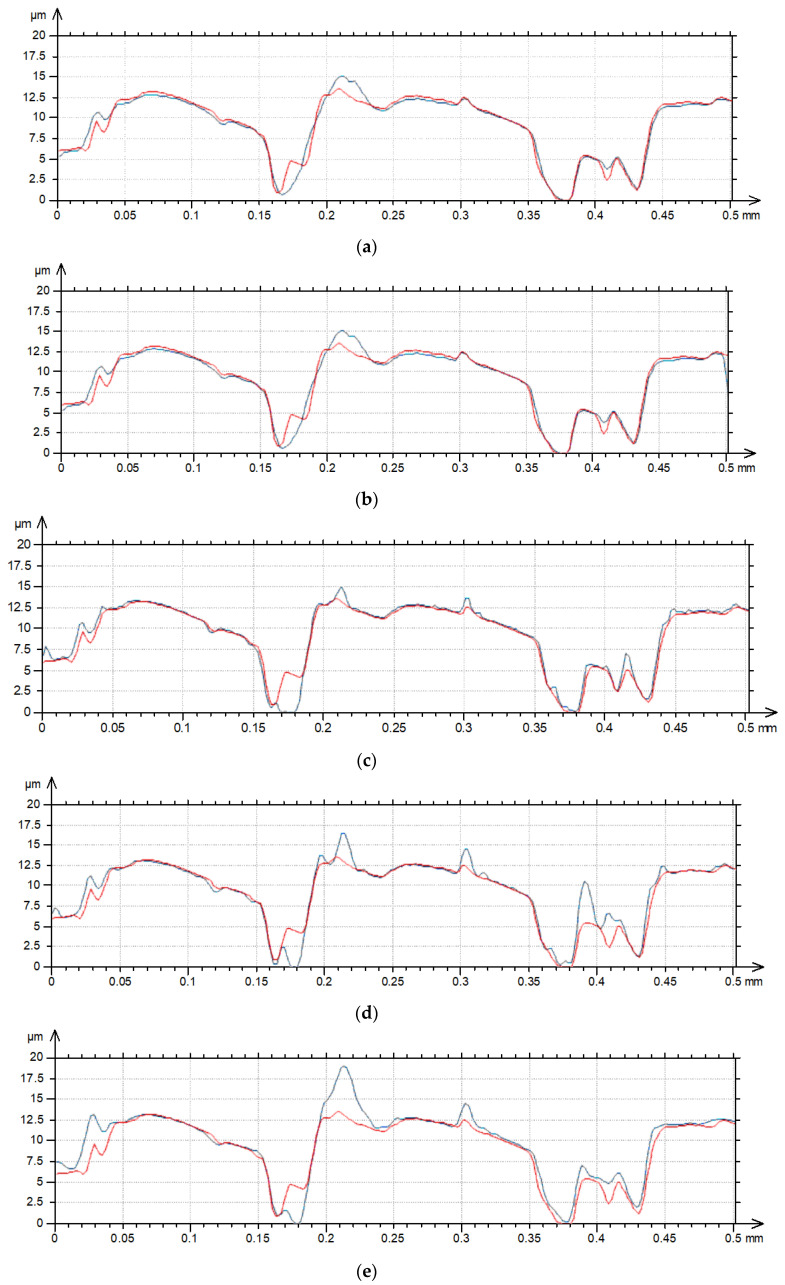
Profile details of surface B after measurements with various speeds: (**a**) 0.5 and 1 mm/s, (**b**) 0.5 and 2 mm/s, (**c**) 0.5 and 3 mm/s, (**d**) 0.5 and 4 mm/s, (**e**) 0.5 and 5 mm/s; red color—0.5 mm/s speed, blue color—higher speeds.

Table 2 presents selected texture parameters of the milled surface B after measurements with various speeds. Figure 4 shows details of the profile of this texture.

The increase in the measuring speed caused small changes in the parameters Sq, Sa, Sv, Ssk, Sku, Sal, and Str of surface B after milling (Table 2). Parameters describing maximum heights Sp and Sz increased with the scanning speed, up to 3 mm/s. The maximum increases in the Sp and Sz parameters were 35% and 21%, respectively. For measuring speeds greater than 1 mm/s, an increase in the peak density occurred of up to 4-fold. The maximum peak density was obtained for a measuring speed of 4 mm/s. Similar changes occurred for the Spc parameter, which increased 2.3-fold.

**Figure 5:**
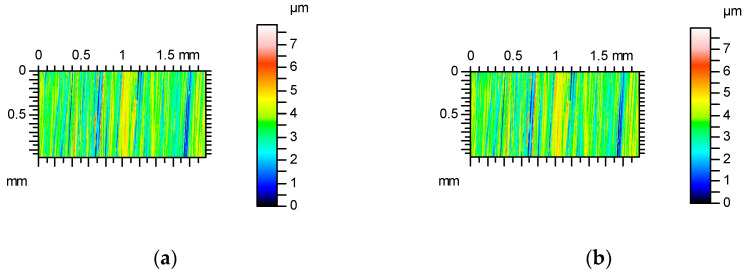

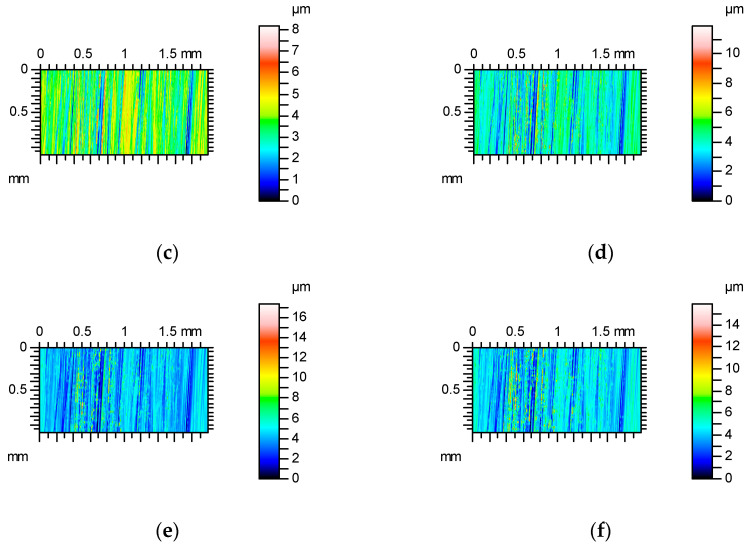
Contour plots of surface C after measurements at various speeds: (**a**) 0.5 mm/s, (**b**) 1 mm/s, (**c**) 2 mm/s, (**d**) 3 mm/s, (**e**) 4 mm/s and (**f**) 5 mm/s.

**Figure 6:**
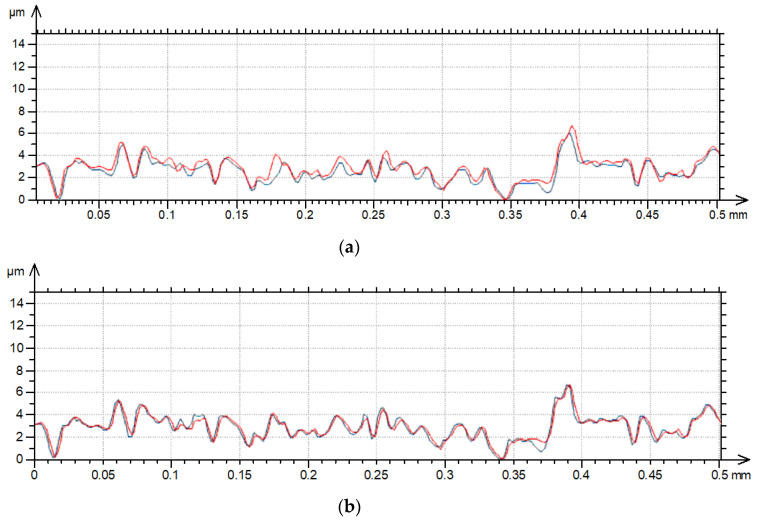

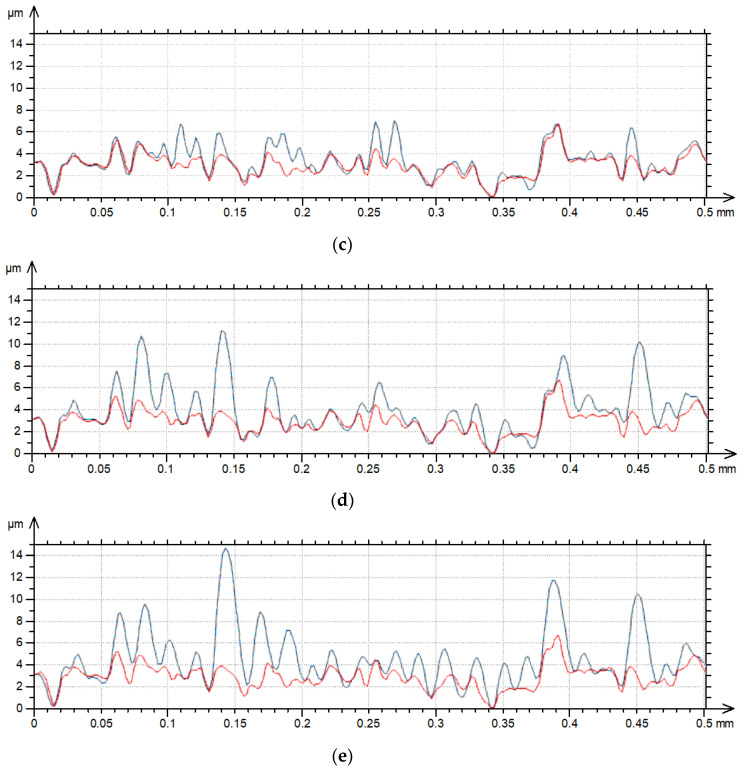
Profile details of surface C after measurements with various speeds: (**a**) 0.5 and 1 mm/s, (**b**) 0.5 and 2 mm/s, (**c**) 0.5 and 3 mm/s, (**d**) 0.5 and 4 mm/s, (**e**) 0.5 and 5 mm/s; red color—0.5 mm/s speed, blue color—higher speeds.

The increase in the measurement speed caused an increase in the hybrid parameters. The changes were small for speeds lower than 2 mm/s, and the highest increases occurred for a measuring speed of 4 mm/s; they were 20% for Sdq and 35% for Sdr. These changes were mostly caused by an increase in the peak density with the traverse speed. The increase in maximum amplitude was evident for measuring speeds of 3 and 4 mm/s. The increase in speed caused an increase in the height of the existing peaks (Figure 4). Between 0.15 and 0.2 mm, the blue curve deviates strongly to smaller values compared to the reference profile taken with 0.5 mm/s. This behavior was probably caused by the sampling interval used. Table 3 presents selected texture parameters of the ground surface C after measurements with various speeds perpendicular to the main direction of the texture (lay). Figure 5 shows contour plots and Figure 6 shows profile details of this texture.

The increase in traverse speed caused changes in all the surface texture parameters analyzed after grinding. Parameters Sq, Ssk, Sku, and Sv increased with scanning speed. The Sz parameter also increased; the greatest change occurred for a speed measurement of 4 mm/s. The spatial parameters Sal and Str decreased with the traverse speed; the greatest changes occurred for the speed of 4 mm/s. The peak density, peak curvature and hybrid parameters increased as the trace speed increased, and the greatest changes occurred for the speed of 3 mm/s. Changes in hybrid parameters are the consequence of changes in surface amplitude parameters and peak density. The maximum changes in parameters were very high; Sq, Sv, Sa, Sdq, Sdr, and Spc increased by 47, 28, 31, 41, 88, and 33%, respectively, while Str and Sal decreased by 53 and 58%, respectively. Kurtosis Sku, Sp, Sz and peak density Spd increased up to 2.4, 3, 2 and 2.8 times, respectively.

The additional peaks that did not actually exist on the surface are visible in the contour plots (Figure 5), especially for traverse speeds on 3, 4 and 5 mm/s. In these cases, the maximum surface height considerably increased, compared to the smallest speed. The height increases of existing peaks are also visible in the profile details (Figure 6); however, additional peaks can also be found.

**Figure 7:**
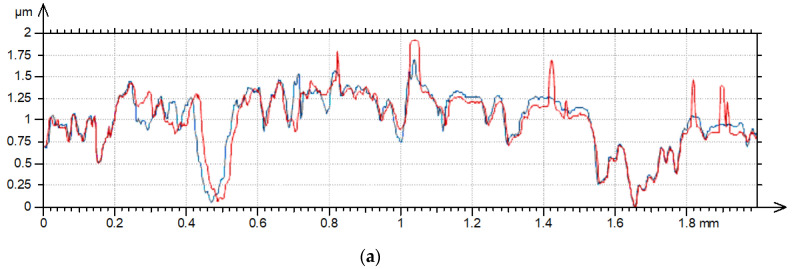

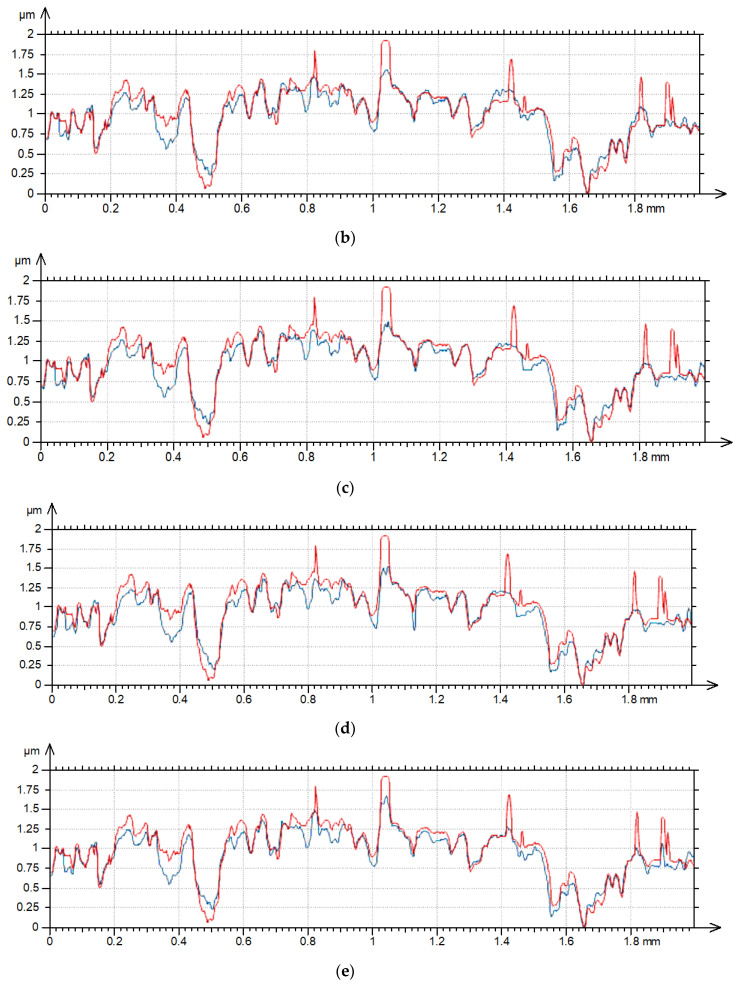
Profile details of surface D after measurements with various speeds: (**a**) 0.5 and 1 mm/s, (**b**) 0.5 and 2 mm/s, (**c**) 0.5 and 3 mm/s, (**d**) 0.5 and 4 mm/s, (**e**) 0.5 and 5 mm/s; red color—0.5 mm/s speed, blue color—higher speeds.

Table 4 presents selected texture parameters of the D ground surface after measurements along the main texture direction (lay) with various speeds. Figure 7 shows details of the profile of this texture.

Surface D was similar to surface C after grinding; however, it was measured along the lay. Profiles of surface D in the measurement direction are characterized by larger main wavelengths and smaller slopes than those of surface C. These changes caused different characteristics of parameter changes with the scanning speed. The average amplitude parameters Sa and Sq had a tendency to decrease with increases in the measurement speed. The peak density Spd typically decreased as the speed increased. The hybrid parameters Sdq, Sdr, and the Spc parameter decreased with increasing measuring speed, up to 10, 25 and 44%, respectively. These reductions are related to decreases in the mean surface height and peak density. The spatial parameters did not show clear changes.

One can see from the analysis of Figure 7 that, differently to the profiles presented in Figure 2, Figure 4 and Figure 6, the heights of some peaks and also the depths of existing valleys decreased. A decrease in height due to the increase in the traverse speed was caused by the relatively low slope in the measurement direction. Please note that the length of the profile shown in Figure 7 is much greater than the lengths of other profiles presented in this article.

**Figure 8:**
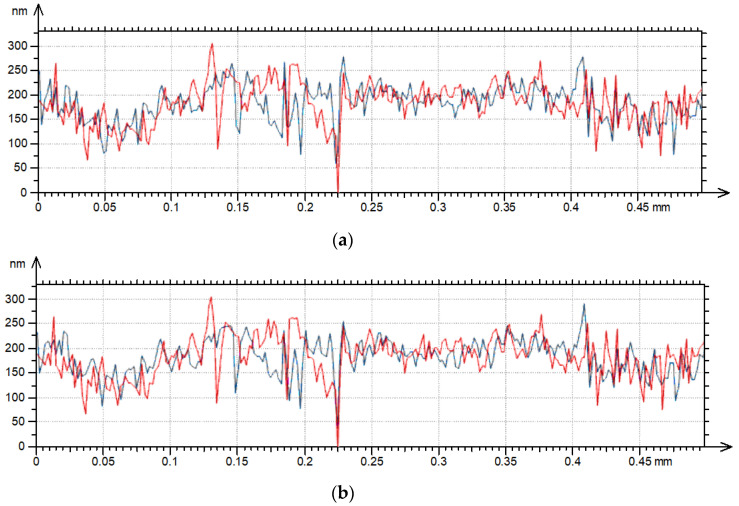

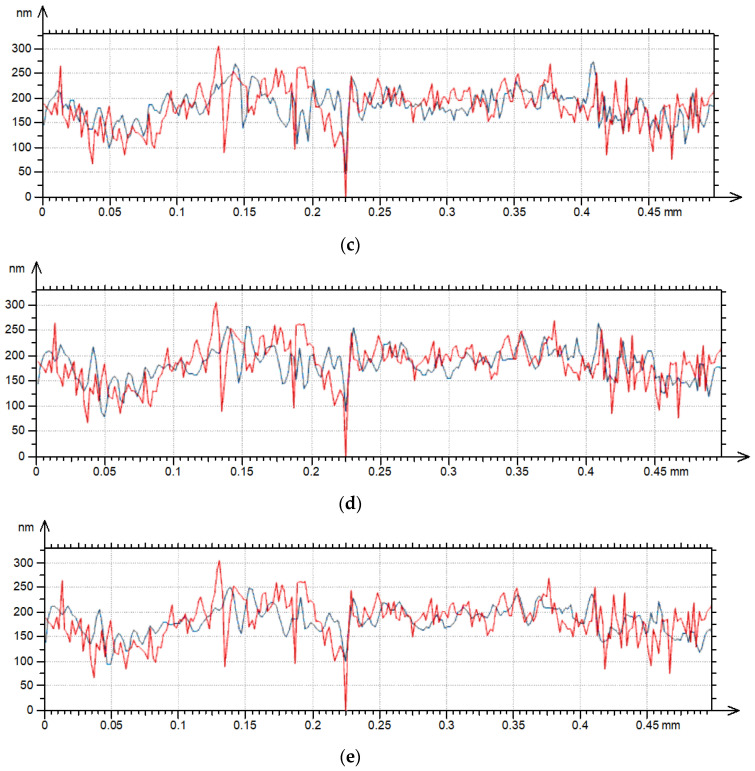
Profile details of surface E after measurements with various speeds: (**a**) 0.5 and 1 mm/s, (**b**) 0.5 and 2 mm/s, (**c**) 0.5 and 3 mm/s, (**d**) 0.5 and 4 mm/s, (**e**) 0.5 and 5 mm/s; red color—0.5 mm/s speed, blue color—higher speeds.

Table 5 presents selected texture parameters of the polished surface E with various speeds. Figure 8 shows the details of the profile of this texture.

The increase in the measurement speed caused increases in the maximum amplitude parameters of the polished surface. The maximum change in the Sp parameter was nearly 3-fold, while in the Sz parameter, it was about 2-fold. The changes in the averaged parameters Sa and Sq were smaller. The increases in Ssk and Sku were also comparatively high. Unlike surfaces analyzed previously, the correlation length Sal increased as a result of the measuring speed’s increase; the greatest increase was nearly 9-fold for the sliding speed of 3 mm/s. Peak density Spd decreased as a result of an increase in the measurement speed of up to 5.4 times. The parameters Str and Spc did not show a clear trend of changes. Hybrid parameters Sdq and Sdr decreased with measuring speed; the greatest reductions were 46 and 77%. This behavior was caused by an increase in the correlation length Sal and a decrease in the peak density Spd.

The profile details obtained for measuring speeds of 3, 4 and 5 mm/s contained a lower amount of high-frequency components (smaller numbers of peaks and valleys are visible) than other profile details presented in Figure 8.

**Figure 9:**
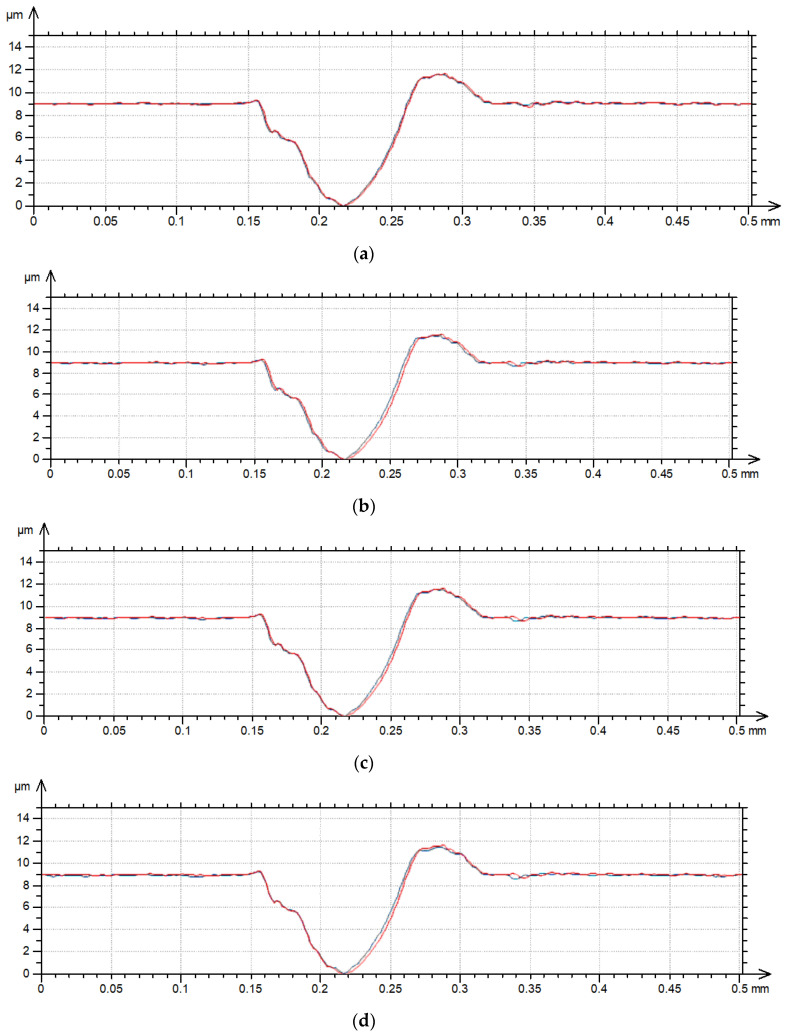

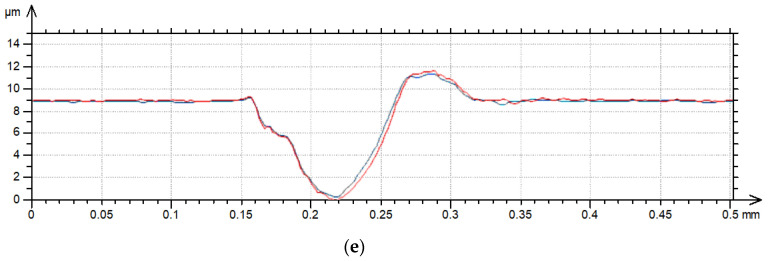
Profile details of surface F after measurements with various speeds: (**a**) 0.5 and 1 mm/s, (**b**) 0.5 and 2 mm/s, (**c**) 0.5 and 3 mm/s, (**d**) 0.5 and 4 mm/s, (**e**) 0.5 and 5 mm/s; red color—0.5 mm/s speed, blue color—higher speeds.

Table 6 presents selected texture parameters of the laser textured surface F after measurements with various speeds. Figure 9 shows the details of the profile of this texture.

The parameters of the laser-textured surface were comparatively stable. Amplitude parameters characterizing maximum height Sp, Sv, and Sz had a tendency to decrease from the scanning speed of 1 mm/s. They are more sensitive to changes in the scanning speed than parameters Sa and Sq. However, the changes in parameters describing maximum height caused by the increase in measuring speed were comparatively small. For example, the maximum decrease in the maximum height Sz was 2%. The tendency of changes in the Spd peak density was unclear. Changes in hybrid parameters caused by increased traverse speed were comparatively high. The maximum decreases in the Sdq and Sdr parameters were 6 and 10%, respectively. The behavior of the Spc parameter was different from those of hybrid parameters. Spc increased when the measuring speed increased to 4 mm/s and then decreased for the highest measuring speed. The highest increase was 44%. Differences in changes in the Spc parameter and hybrid parameters were probably caused by the stratified character of the laser-textured surface.

The details of the flattened profile outside the oil pocket and the reduction in the oil pocket depth are visible in Figure 9.

**Figure 10:**
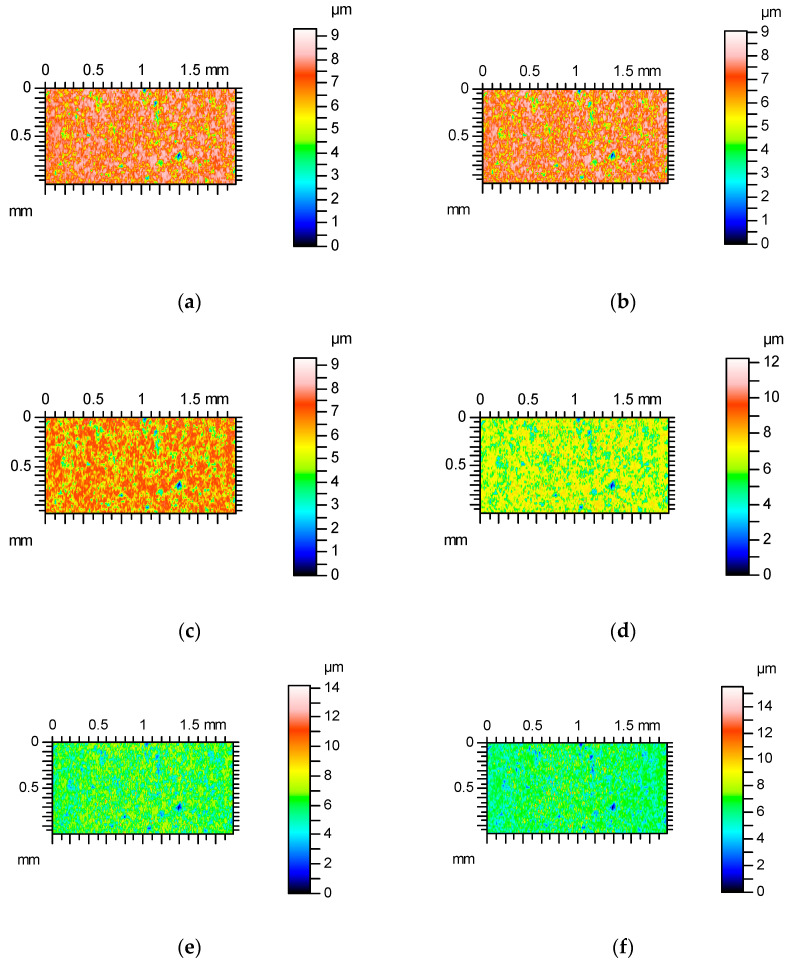
Contour plots of surface G after measurements with various speeds: (**a**) 0.5 mm/s, (**b**) 1 mm/s, (**c**) 2 mm/s, (**d**) 3 mm/s, (**e**) 4 mm/s and (**f**) 5 mm/s.

**Figure 11:**
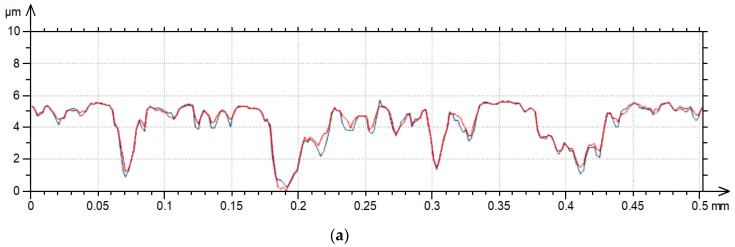

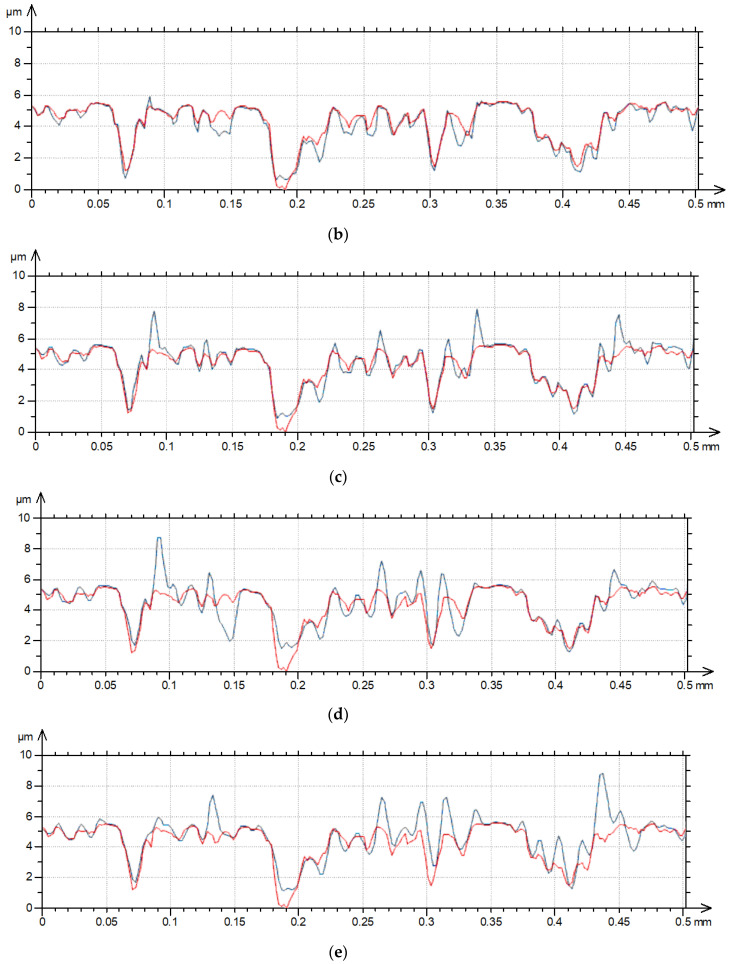
Profile details of surface G after measurements with various speeds: (**a**) 0.5 and 1 mm/s, (**b**) 0.5 and 2 mm/s, (**c**) 0.5 and 3 mm/s, (**d**) 0.5 and 4 mm/s, (**e**) 0.5 and 5 mm/s; red color—0.5 mm/s speed, blue color—higher speeds.

Table 7 presents selected texture parameters of the two-process random surface G after measurements with various speeds. Figure 10 shows contour plots and Figure 11 shows profile details of this texture.

The analyzed surface G was created by abrasive blasting followed by lapping. The increase in traverse speed caused an increase in amplitude of the surface. Only the Sv parameter decreased, to 14%. The increases in the averaged parameters were comparatively small—of Sq to 13% and Sa to 9%. However, the parameters Sz and Sp increased more—1.7 and 4.3 times, respectively. A large change in the Sp parameter was related to a large increase in the emptiness coefficient Sp/Sz [28] from 0.3 to 0.6. This is related to an increase in the skewness Ssk from −1.47 to −0.06. Due to an increase in measuring speed from 0.5 to 5 mm/s, two-process surfaces looked like one-process surfaces. Even an increase in the scanning speed to 1 mm/s caused significant changes in Ssk. Due to an increase in the measuring speed to 4 mm/s, the Sku kurtosis increased and then decreased for the highest speed. The increase in the traverse speed caused decreases in the spatial parameters Sal and Str of up to 44 and 20%, respectively, while the peak density Spd increased up to 2.6 times. Changes in surface amplitude and mean wavelength led to increases in hybrid parameters Sdq and Sdr of up to 21 and 60%, respectively. The behavior of the peak’s mean curvature Spc was similar to changes in hybrid parameters; the highest increase in Spc due to increases in the tracing speed were 2.9 times and occurred for the speed of 3 mm/s. In general, changes in the analyzed parameters of surface G were comparatively high.

The analyses of the contour plots from Figure 10 prove increases in height and peak density when measuring speed increased, particularly for measuring speeds of 3, 4 and 5 mm/s. The increase in the heights of the existing peaks and the formation of additional peaks due to the increase in the traverse speed are visible in Figure 11.

Because the surfaces presented were representative for the machining processes, similar results were obtained for other surfaces studied.

The increase in measuring speed caused an increase in the roughness height and peak density of the surfaces after abrasive blasting. These changes caused increases in the hybrid parameters Sdq, Sdr, and peak density Spd; the relative changes in these parameters were comparatively high.

Changes in surface parameters after milling caused by the increase in traverse speed were relatively low. For rougher surfaces characterized by an Sq parameter greater than 1.5 µm, due to the increase in the scanning speed, the peak density and surface height increased; however, the change in amplitude was low. These deviations caused an increase in the parameters Sdq, Sdr, and Spc. Different results were given for smoother surfaces. The decrease in the height of the roughness or an increase in the correlation length caused a decrease in hybrid parameters, although the peak density and mean peak curvature were seen to increase.

The tendencies of changes in the parameters of the ground surfaces depended on the direction of measurement. When the surfaces were measured perpendicular to the lay (main texture direction), the roughness height and peak density increased, while the correlation length decreased. Changes in amplitude and main wavelengths caused increases in hybrid parameters and the mean peak curvature. These changes were comparatively high, even for comparatively smooth surfaces (Sq about 0.5 µm). When surfaces were measured along the lay, the hybrid parameters and Spc decreased; this behavior was related to decreases in peak density and averaged roughness height, although the maximum amplitude could increase and correlation length could decrease with traverse speed.

The hybrid parameters Sdq and Sdr of smooth surfaces after polishing or lapping decreased with scanning speed. This behavior was connected with an increase in correlation length Sal, a decrease in peak density Spd, and/or a decrease in roughness height.

Changes of parameters of laser textured surfaces due to the increase in the roughness height mainly depended on the surface details free of dimples. They were machined by lapping/polishing or grinding; therefore, the hybrid parameters could increase or decrease. The effects of oil pockets were less notable, because the pit–area ratio was less than 20%. However, the presence of dimples typically caused smaller changes in parameters compared to untextured surfaces. Changes in the values of the Sal parameter were low.

The hybrid parameters of the two-process random textures increased, which was related to an increase in height, a decrease in the correlation length, and an increase in the peak density. In most cases, the parameters characterizing the shape of the ordinate distribution Sp/Sz or Ssk changed, and the two-process surface measured with high speed looked similar to the one-process surface. This behavior was connected with creation of high peaks that did not really exist on the surfaces.

**Figure 12:**
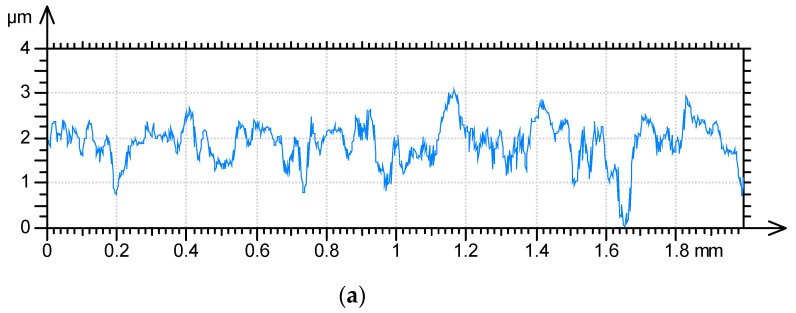

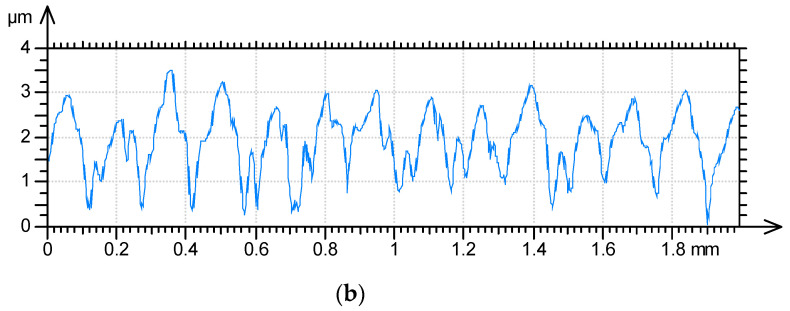
Profiles after grinding (**a**) and milling (**b**) characterized by the Pdq parameter of 3.6; the PSm of the ground profile was 0.055 mm, the PSm of the milled profile was 0.13 mm.

**Figure 13:**
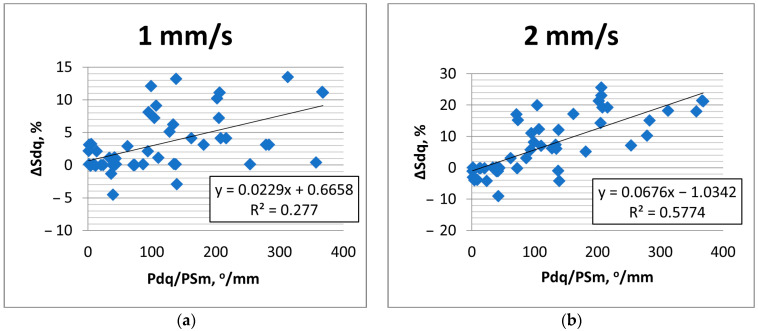

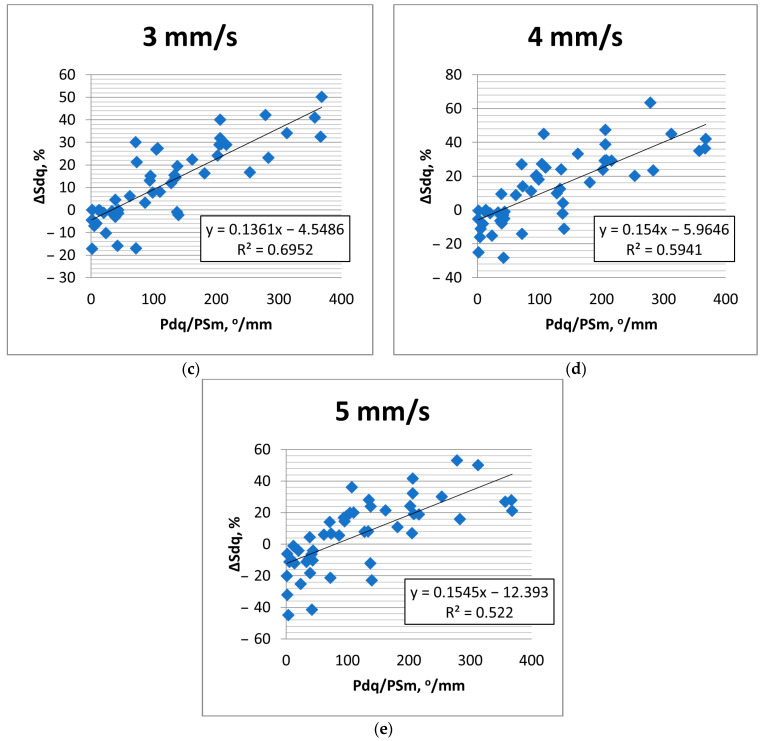
Dependence between the Pdq/PSm ratio and the relative change in the Sdq parameter caused by the increase in the traverse speed from: (**a**) 0.5 mm/s to 1 mm/s, (**b**) 2 mm/s, (**c**) 3 mm/s, (**d**) 4 mm/s and (**e**) 5 mm/s.

The effect of the increase in the traverse speed caused an increase in the heights of new peaks, an increase in the heights of existing peaks, or the flattening of the surface due to the decrease in existing valleys. The first two behaviors are related to the increase in the hybrid parameters, and the third to the decrease in the hybrid parameters. Sometimes all the behaviors mentioned existed. Changes in hybrid parameters are related to changes in amplitude, peak density, or correlation length. High changes in hybrid parameters due to the increase in the measuring speed were also found in the technical literature [9,10,12]. From the hybrid parameters, rms. slope Sdq was found to be more important than the Sdr parameter. For a relatively high sampling interval, the parameters Sdq and Sdr are interrelated [25]. Sdr is more sensitive to measurement errors than Sdq [29]. Therefore, the change of rms. slope due to the increase in measurement speed was analyzed in detail. An increase in Sdq with the measurement speed was found for surface textures characterized by a large slope. For smoother textures, typically, the rms. slope decreased with the traverse speed. Therefore, the change in the Sdq parameter depended on the initial rms. slope Pdq of the measured profile. The higher the profile slope, the greater the possibilities of creating more peaks or increases in peak heights. The flattening of the surface is more possible for smoother surfaces with low slope Pdq. However, it was also found that for surface profiles characterized by a similar slope (Figure 12), the parameters’ changes were greater for profiles with smaller values of the horizontal parameter PSm (mean width of the elements of the profile). The change in slope Sdq of the surface texture with the profile shown in Figure 12a (after grinding) was much greater than that shown in Figure 12b (after milling). The importance of the PSm parameter could be related with the possibilities of creating more additional (non-existing) peaks or the increasing heights of more peaks on the surface characterized by a smaller value of this parameter. Due to the decrease in PSm, stylus flight can occur more frequently. Therefore, the change in the rms. slope Sdq with the measuring speed can be related to the parameter Pdq/PSm (°/mm). Because the pit–area ratios of laser-textured surfaces were smaller than 20%, profile parameters of these surfaces were obtained from surface details free of dimples. Figure 13 presents the result of this analysis. The values of the parameters Pdq and PSm were obtained as average values of profiles measured in the measurement direction for a traverse speed of 0.5 mm/s; the sampling interval was 2 µm, and the evaluation length was 2 mm.

The relative change in the Sdq parameter due to the increase in the measured speed was positive when the Sdq parameter increased and negative when it decreased. The coefficient of determination R^2^ was used to describe the statistical connection between Pdq/PSm and ΔSdq. These parameters were assumed to be highly correlated when R^2^ was greater than 0.5. In this case, 50% of the variability of ΔSdq was caused by the Pdq/PSm of the profile measured with 0.5 mm/s speed.

This relation was not strong for the measuring speed of 1 mm/s. However, in this case, the relative error of rms. slope Sdq determination was small—the maximum increase was less than 14%, while the maximum decrease was less than 5%, and the average error was 3.7%. However, the analyzed relation was strong for higher measurement speeds. When the measuring speed was 2 mm/s, the maximum increase in Sdq was about 25%, while the maximum decrease was about 9%, and the average error was 8%. Relative changes in parameters were higher for the traverse speed of 3 mm/s. In this case, the dependence between the variables analyzed was the strongest. The maximum increase in Sdq was 50%, but the maximum decrease was 18%; the average relative change was 15.5%. Higher changes in rms. slope Sdq were obtained due to the increase in the measurement speed to 4 mm/s. The maximum increase in Sdq was 60%, but the maximum decrease was 30%; the average relative change was 19%. For the highest measurement speed of 5 mm/s, the increase in the Sdq parameter was smaller compared to those obtained for a measuring speed of 4 mm/s. The maximum increase was 53%. However, the decreases in the Sdq parameter were the highest out of all analyzed cases; the maximum decrease was 45%. The average change in the Sdq parameter was similar to that obtained for the measuring speed of 4 mm/s and amounted to 19%. The highest speed used changed the surface texture more than a speed of 4 mm/s. For all measurement speeds, typically, decreases in ΔSdq appeared for profiles with Pdq/PSm smaller than 50°/mm, but other cases were also possible.

A similar relation between the parameter characterizing the surface profile along the measurement direction and changes in rms. slope Sdq owing to a growth in the measuring speed has not yet been found. The slope of the surface is tribologically important, related to friction, wear, and the capacity for plastic deformation [25,30,31]. However, it is sensitive to minor changes in the measuring system. In particular, the slope is highly affected by high-frequency noise [32,33] and depends on the sampling interval [29]. Therefore, we must be cautious when using the surface slope for any application. However, for the same stylus tip profilometer, evaluation area, sampling interval, measuring speed and stylus force, the results regarding variations in the rms. slope in repetitive measurements were much smaller than those regarding the change due to the increase in the traverse speed.

## 4. Conclusions

1. Changes in the surface topography parameters due to increase in traverse speed of the stylus tip depend substantially on the characteristics of the surfaces and the values of parameters.
2. The increase in the speed of measurement caused changes in surface amplitude, correlation length Sal, mean summit curvature, peak density, and hybrid parameters. Changes in parameters related to the peaks and hybrid parameters were the largest. The changes in Sdr are greater than the changes in rms. slope Sdq.
3. The errors in the determination of hybrid parameters depend on changes in amplitude and spatial parameters. In general, parameters of rougher surfaces or of surfaces characterized by small wavelengths changed differently from smooth surfaces or surfaces with large main wavelengths. For the first group of surfaces, increases in traverse speed caused increases, but for the last group, decreases in hybrid parameters were seen.
4. The hybrid parameters of the surfaces after abrasive blasting and of the random two-process surfaces after abrasive blasting and lapping increased with the traverse speed. These parameters of the surfaces after polishing and lapping decreased due to the increase in the scanning speed. The increase in the velocity of the measurement caused notable changes in the parameters that describe the shape of the ordinate distribution of random two-process surfaces. Changes in surface parameters after milling and grinding due to increase in traverse speed depended on the surface amplitude and direction of the measurements.
5. The increase in the measuring speed from 0.5 to 1 mm/s caused small changes in the parameters. The maximum increase in the Sdq parameter was 14%, but the maximum decrease was 5%.
6. The increase in measurement speed caused an increase in heights of existing peaks and the formation of new peaks that did not really exist on the first side, as well as a decrease in depths of valleys and surface flattening on the second side. The first possibility led to an increase in slope, while the second caused a decrease in slope.
7. An increase in the traverse speed usually caused greater changes in surface topography parameters. Relative slope changes were proportional to the Pdq/PSm ratio of the profile measured with a speed of 0.5 mm/s. Typically, a decrease in ΔSdq appeared for Pdq/PSm less than 50°/mm. Measurement with a speed of 5 mm/s led to a smaller decrease in rms. slope Sdq compared to the measurement with a speed of 4 mm/s.
8. In future work, similar research will be carried out using samples subjected to other machining and wear processes made from different materials. The impact of the flight of the stylus will be simulated.

## Figures and Tables

**Table 1:** Selected texture parameters of surface A after measurements with various speeds.

Parameter/Speed, mm/s	0.5	1	2	3	4	5
Sq, µm	2.05 (0.003)	2.06 (0.004)	2.08 (0.008)	2.18 (0.009)	2.31 (0.02)	2.33 (0.01)
Ssk	0.04 (0.02)	−0.04 (0.02)	−0.07 (0.03)	−0.04 (0.04)	0.13 (0.09)	0.33 (0.07)
Sku	6.42 (0.1)	6.53 (0.11)	6.52 (0.14)	6.19 (0.18)	5.99 (0.23)	5.98 (0.1)
Sp, µm	14.4 (0.04)	14.5 (0.05)	14.3 (0.11)	14.2 (0.13)	14.7 (0.3)	15.9 (0.09)
Sv, µm	11.7 (0.07)	11.9 (0.1)	12 (0.04)	14.1 (0.05)	14.6 (0.07)	13.6 (0.06)
Sz, µm	26.1 (0.15)	26.4 (0.2)	26.3 (0.15)	28.3 (0.22)	29.3 (0.5)	28.5 (0.15)
Sa, µm	1.41 (0.001)	1.41 (0.001)	1.43 (0.012)	1.52 (0.013)	1.61 (0.02)	1.63 (0.001)
Sal, mm	0.022 (5 × 10^−5^)	0.022 (5 × 10^−5^)	0.022 (8 × 10^−5^)	0.022 (8 × 10^−5^)	0.022 (0.00017)	0.022 (0.00019)
Str	0.78 (0.001)	0.77 (0.0011)	0.77 (0.005)	0.78 (0.0055)	0.78 (0.009)	0.78 (0.005)
Sdq	0.31 (0.001)	0.32 (0.0012)	0.34 (0.008)	0.37 (0.01)	0.37 (0.01)	0.32 (0.001)
Sdr, %	4.45 (0.008)	4.56 (0.008)	5.23 (0.22)	6.23 (0.24)	6.25 (0.25)	5.26 (0.01)
Spd, 1/mm^2^	325 (4.1)	333 (5.1)	369 (12.6)	464 (35.1)	451 (46.1)	410 (5.2)
Spc, 1/mm	169 (2.1)	182 (2.9)	224 (4.8)	225 (12.5)	226 (13.2)	166 (2.2)

**Table 2:** Selected texture parameters of surface B after measurements with various speeds.

Parameter/Speed, mm/s	0.5	1	2	3	4	5
Sq, µm	4.37	4.4	4.41	4.41	4.42	4.38
Ssk	−0.49	−0.49	−0.5	−0.5	−0.48	−0.48
Sku	2.26	2.26	2.25	2.3	2.36	2.35
Sp, µm	13.9	13.9	13.9	18.8	17.3	15.8
Sv, µm	10.6	10.7	10.7	10.9	10.9	10.8
Sz, µm	24.5	24.6	24.6	29.7	28.2	26.6
Sa, µm	3.76	3.79	3.8	3.77	3.76	3.73
Sal, mm	0.04	0.039	0.039	0.039	0.039	0.039
Str	0.079	0.079	0.079	0.078	0.078	0.078
Sdq	0.35	0.35	0.36	0.39	0.42	0.37
Sdr, %	5.27	5.36	5.65	6.48	7.13	5.96
Spd, 1/mm^2^	82.7	74.6	98.8	206.6	336	258.8
Spc, 1/mm	143	105	243	329	274	205

**Table 3:** Selected texture parameters of surface C after measurements with various speeds.

Parameter/Speed, mm/s	0.5	1	2	3	4	5
Sq, µm	0.95	0.98	1	1.18	1.35	1.4
Ssk	−0.28	−0.34	−0.32	0.36	0.97	1.2
Sku	3.45	3.56	3.54	5.08	7.58	8.18
Sp, µm	4.42	4.47	4.63	8.03	13.11	11.54
Sv, µm	3.42	3.52	3.55	3.86	4.25	4.38
Sz, µm	7.84	7.97	8.18	11.89	17.38	15.92
Sa, µm	0.75	0.77	0.79	0.88	0.97	0.98
Sal, mm	0.021	0.021	0.018	0.01	0.0088	0.0093
Str	0.041	0.042	0.035	0.02	0.019	0.02
Sdq	0.22	0.22	0.26	0.31	0.3	0.29
Sdr, %	2.35	2.53	3.21	4.41	4.25	3.72
Spd, 1/mm^2^	149	158	336	422	352	341
Spc, 1/mm	165	169	260	220	164	140

**Table 4:** Selected texture parameters of surface D after measurements with various speeds.

Parameter/Speed, mm/s	0.5	1	2	3	4	5
Sq, µm	0.44	0.43	0.43	0.43	0.43	0.43
Ssk	−0.45	−0.35	−0.37	−0.41	−0.42	−0.4
Sku	3.14	3.06	2.99	3.09	3.07	3.08
Sp, µm	1.55	1.38	1.38	1.44	1.6	1.95
Sv, µm	2.2	2.31	2.41	2.2	2.3	2.29
Sz, µm	3.75	3.69	3.79	3.64	3.9	4.24
Sa, µm	0.35	0.35	0.35	0.34	0.34	0.34
Sal, mm	0.033	0.035	0.037	0.032	0.033	0.033
Str	0.033	0.035	0.037	0.032	0.032	0.033
Sdq	0.049	0.049	0.047	0.046	0.045	0.044
Sdr, %	0.12	0.12	0.11	0.11	0.1	0.09
Spd, 1/mm^2^	219	221	213	224	202	183
Spc, 1/mm	25	24	19	18	16	14

**Table 5:** Selected texture parameters of surface E after measurements with various speeds.

Parameter/Speed, mm/s	0.5	1	2	3	4	5
Sq, µm	0.041	0.041	0.044	0.047	0.04	0.037
Ssk	−0.53	−0.6	−0.32	0.03	0.023	0.17
Sku	5.76	7.02	6.74	6.43	10.65	9.91
Sp, µm	0.31	0.31	0.38	0.53	0.97	0.91
Sv, µm	0.61	0.78	0.85	0.85	0.72	0.45
Sz, µm	0.92	1.09	1.23	1.28	1.69	1.36
Sa, µm	0.03	0.03	0.033	0.036	0.029	0.027
Sal, mm	0.0091	0.0081	0.03	0.081	0.015	0.011
Str	0.22	0.38	0.03	0.08	0.25	0.27
Sdq	0.023	0.023	0.022	0.019	0.015	0.012
Sdr, %	0.03	0.03	0.024	0.019	0.012	0.007
Spd, 1/mm^2^	1392	1115	756	603	259	280
Spc, 1/mm	21.3	24.3	27.1	25.3	23.2	14.5

**Table 6:** Selected texture parameters of surface F after measurements with various speeds.

Parameter/Speed, mm/s	0.5	1	2	3	4	5
Sq, µm	1.46	1.46	1.46	1.46	1.45	1.45
Ssk	−3.15	−3.16	−3.2	−3.16	−3.2	−3.2
Sku	14.6	14.6	14.8	14.6	14.8	14.8
Sp, µm	5.76	5.69	5.65	5.36	5.61	5.53
Sv, µm	9.32	9.51	9.29	9.17	9.29	9.24
Sz, µm	15.08	15.2	14.94	14.53	14.9	14.77
Sa, µm	0.75	0.75	0.75	0.75	0.75	0.75
Sal, mm	0.045	0.045	0.045	0.045	0.045	0.045
Str	0.82	0.83	0.82	0.83	0.82	0.82
Sdq	0.089	0.089	0.088	0.086	0.085	0.084
Sdr, %	0.39	0.39	0.38	0.37	0.36	0.35
Spd, 1/mm^2^	16.6	16.1	16.1	17.6	16.6	15.1
Spc, 1/mm	43.2	43.1	46.8	58.2	62.4	44.8

**Table 7:** Selected texture parameters of the surface G after measurements with various speeds.

Parameter/Speed, mm/s	0.5	1	2	3	4	5
Sq, µm	1	1	1	1.04	1.08	1.13
Ssk	−1.47	−1.26	−1.12	−0.9	−0.42	−0.06
Sku	5.85	4.93	4.36	4.21	4.3	5.14
Sp, µm	2.15	2.17	3.03	5.81	8	9.22
Sv, µm	7.18	6.88	6.29	6.44	6.16	6.25
Sz, µm	9.33	9.05	9.32	12.25	14.16	15.47
Sa, µm	0.78	0.8	0.8	0.82	0.84	0.85
Sal, mm	0.025	0.023	0.022	0.019	0.016	0.014
Str	0.64	0.64	0.63	0.63	0.57	0.51
Sdq	0.19	0.2	0.2	0.24	0.24	0.24
Sdr, %	1.77	2.04	1.99	2.83	2.75	2.66
Spd, 1/mm^2^	185	253	261	459	486	469
Spc, 1/mm	66	76	78	194	150	136

## Data Availability

The original contributions presented in the study are included in the article, further inquiries can be directed to the corresponding author.
